# Advancing Non-Small-Cell Lung Cancer Management Through Multi-Omics Integration: Insights from Genomics, Metabolomics, and Radiomics

**DOI:** 10.3390/diagnostics15202586

**Published:** 2025-10-14

**Authors:** Martina Pierri, Giovanni Ciani, Maria Chiara Brunese, Gianluigi Lauro, Stefania Terracciano, Maria Iorizzi, Valerio Nardone, Maria Giovanna Chini, Giuseppe Bifulco, Salvatore Cappabianca, Alfonso Reginelli

**Affiliations:** 1 Department of Pharmacy, University of Salerno, Via Giovanni Paolo II, 132, 84084 Fisciano, Italy; mpierri@unisa.it (M.P.); glauro@unisa.it (G.L.); bifulco@unisa.it (G.B.); 2 Department of Precision Medicine, University of Campania “L. Vanvitelli”, 80138 Naples, Italy; dott.giovanniciani@gmail.com (G.C.); mariachiarabrunese@gmail.com (M.C.B.); valerio.nardone@unicampania.it (V.N.); salvatore.cappabianca@unicampania.it (S.C.); alfonso.reginelli@unicampania.it (A.R.); 3 Department of Biosciences and Territory, University of Molise, Contrada Fonte Lappone, 86090 Pesche, Italy; iorizzi@unimol.it

**Keywords:** non-small-cell lung cancer, genomics, metabolomics, radiomics, biomarkers, molecular diagnostics

## Abstract

The integration of multi-omics technologies is transforming the landscape of cancer management, offering unprecedented insights into tumor biology, early diagnosis, and personalized therapy. This review provides a comprehensive overview of the current state of omics approaches, with a particular focus on the application of genomics, NMR-based metabolomics, and radiomics in non-small cell lung cancer (NSCLC). Genomics currently represents one of the most established omics technologies in oncology, as it enables the identification of genetic alterations that drive tumor initiation, progression, and therapeutic response. Interestingly, genomic analyses have revealed that many tumors harbor mutations in genes encoding metabolic enzymes, thus establishing a tight connection between genomics and tumor metabolism. In parallel, metabolomics profiling—by capturing the metabolic phenotype of tumors—has, in recent years, identified specific biomarkers associated with tumor burden, progression, and prognosis. Such findings have catalyzed growing interest in metabolomics as a complementary approach to better characterize cancer biology and discover novel diagnostic and therapeutic targets. Moreover, radiomics, through the extraction of quantitative features from standard imaging modalities, captures tumor heterogeneity and contributes predictive information on tumor biology, treatment response, and clinical outcomes. As a non-invasive and widely available technique, radiomics has the potential to support longitudinal monitoring and individualized treatment planning. Both metabolomics and radiomics, when integrated with genomic data, could support a more comprehensive understanding of NSCLC and pave the way for the development of non-invasive, predictive models and personalized therapeutic strategies. In addition, we explore the specific contributions of these technologies in enhancing clinical decision-making for lung cancer patients, with particular attention to their potential in early diagnosis, treatment selection, and real-time monitoring.

## 1. Introduction

Over the past few years, high-yielding and innovative Omics technologies have shown their revolutionary contribution to scientific progress through the identification of novel biomarkers and molecular profiling in a specific cellular context, proving great potential in generating new clinical insights and leading to the so-called “multi-omics” era [1]. Omics technology refers to a set of scientific techniques and approaches that aim to comprehensively study and analyze various biological molecules or components within a specific biological system. The term “Omics” is derived from the suffix of each specific technique, and it refers to an approach capable of generating a complete set of data by examining all relevant components [2]. Nowadays, several Omics techniques are gaining increasing attention from the scientific community due to their versatility in focusing on different aspects of biological molecules. In fact, through the use of high-throughput screening methods such as DNA sequencing and mass spectrometry, these technologies are capable of generating large-scale datasets that enable comprehensive biological analysis in a relatively short time [3]. Managing and analyzing the vast amounts of data generated in clinical research and clinical practice requires the use of advanced computational and bioinformatics tools, which are fundamental for summarizing and interpreting the results [4]. Overall, Omics has revolutionized our understanding of biology, disease mechanisms, and drug development, enabling scientists to explore biological systems in unprecedented detail.

A crucial role is played by Omics, particularly in cancer research and treatment, since it provides a comprehensive view of the molecular and genetic changes associated with tumors. Importantly, these technologies enable the identification of potential biomarkers for early detection, support the prediction of patient outcomes, and aid the development of increasingly tailored and efficient therapies, providing valuable insights into cancer mechanisms not only for scientists but also for clinicians in their understanding and decision-making [5]. At present, different Omics technologies play a crucial role in cancer:

- Genomics is the study of the entire genome. It involves sequencing, mapping, and analyzing genetic material to understand the function of specific genes in diseases, identify genetic variations, and explore genetic relationships. Specifically in cancer, genomics analysis focuses on identifying mutations, copy number alterations, and structural variations in the genome. This information helps to identify driver mutations and potential therapeutic targets [6].
- Transcriptomics involves the study of all the RNA transcripts of a cell, hence focusing on the study of gene expression patterns. This technology is particularly useful to understand which genes are actively expressed in a specific disease and how gene expression patterns change under different conditions. Transcriptomics is particularly useful in cancer since it provides insights into tumor cells, helping to identify aberrant transcriptions and the corresponding genes that are actively involved in the disease. This information helps researchers to understand molecular pathways involved in cancer development and progression, but also in the discovery of new molecular targets for anticancer therapies [7].
- Proteomics involves the study of the complete set of proteins in a cell or tissue. It involves the identification, quantification, and characterization of proteins to understand their functions, interactions, and modifications. More specifically, in cancer research, proteomics can identify changes in protein expression levels, post-translational modifications, and protein–protein interactions. This information is valuable for understanding the functional consequences of genomic alterations that lead to tumor growth and metastasis. Importantly, proteomics contributed to the identification of clinical biomarkers as well as new therapeutic targets [8].
- Metabolomics analyzes small-molecule metabolites within a cell, and it can provide critical information about the state. In fact, changes in metabolic pathways are often associated with cancer, and metabolites in biological samples can help to identify new biomarkers for cancer diagnosis, monitoring, and therapy [9].
- Epigenomics investigates changes due to the intricate set of epigenetic modifications, such as DNA methylation and histone modification. A biological picture given by epigenomics can help scientists correlate epigenetic changes in cancer development and progression [10].
- Pharmacogenomics combines genomics and pharmacology to study how an individual’s genetic makeup influences the response to drugs. Especially in cancer, it helps in personalized medicine by tailoring treatments for a specific patient’s genetic profile, making therapies safer and more effective. In fact, although cancers may have specific disease-defining mutations, a patient’s genetic variation could affect drug response [11].
- Radiomics is the analysis of the numerical features extracted by radiological images. It converts the qualitative information from the images into numbers, which are invisible to the naked eye view. Radiomics feature extraction can be performed from all imaging modalities such as Computed Tomography (CT), Magnetic Resonance Imaging (MRI), and ultrasound (US), among others. Feature selection and radiomic analysis, instead, require a machine learning model or a formal analysis [12,13].

**Figure 1:**
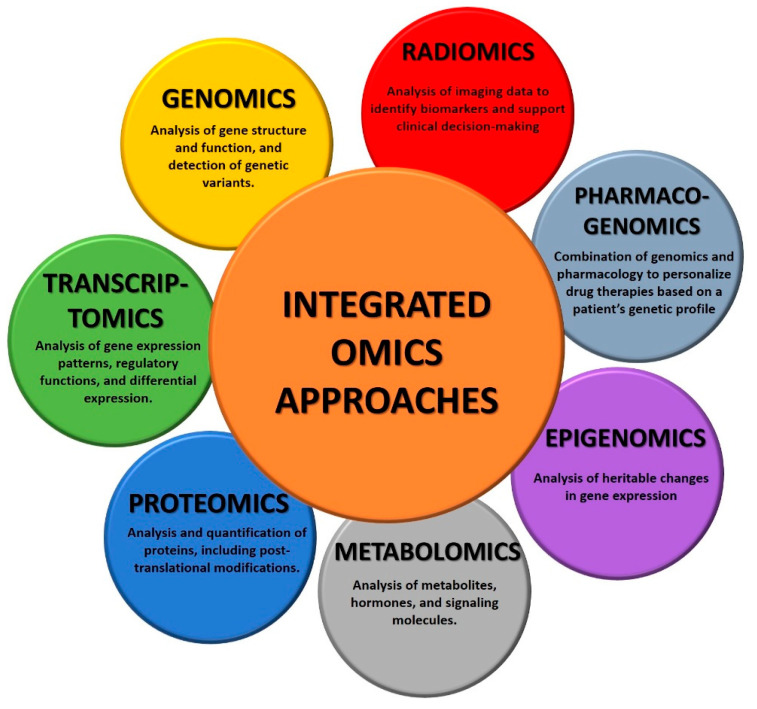
Overview of integrated omics technologies.

The integration of data derived from multiple Omics disciplines could provide a holistic understanding of cancer biology and guide personalized treatment strategies, ultimately improving cancer diagnosis and management (Figure 1).

In this review, we provide an overview of current and future opportunities of Omics technologies in cancer. In particular, the attention will be focused on the application of genomics, metabolomics, and radiomics in lung cancer (LC), which still represents the most lethal type of tumor with the highest mortality rate [14]. In fact, with an incidence of more than two million new cases per year, lung cancer represents the most commonly diagnosed cancer worldwide in males (1.57 million new cases) and the second most diagnosed cancer in females (0.91 million new cases), and is the leading cause of cancer-related deaths globally [15]. Lung cancer is classified as small-cell lung cancer (SCLC) or non-small-cell lung cancer (NSCLC) based on distinct biological characteristics: among them, NSCLC accounts for over 80% of all cases, most of which are diagnosed at advanced stages [16]. For early-stage and operable NSCLC (Stages I and II), the average 5-year survival rate is between 50 and 70%. However, for individuals where tumors have metastasized, the five-year survival rate drops to 30% in Stage III with lymph node involvement and falls to 2–5% in Stage IV metastatic disease [17,18]. The high death rate from lung cancer is a result of the difficulty in making a precise and prompt early diagnosis [19,20].

Despite advances in targeted therapies and immunotherapy, prognosis remains difficult at advanced stages [21]. Symptoms are currently the main basis for diagnosis, and cancer detection frequently happens at a late stage, leading to a very poor prognosis. The overall morbidity for this disease might be significantly changed if the diagnosis could be moved to the early stages. Therefore, the development and improvement of minimally invasive or non-invasive screening platforms—including genomics, metabolomics and radiomics—for the early detection of new biomarkers represent a huge step forward in cancer diagnosis and prognosis, especially for lung cancer, which may have subclinical symptoms for years before clinical manifestation.

## 2. Role of Genomics in Cancer

Nowadays, genomics represents one of the primary Omics technologies employed for understanding cancer, as it enables researchers and clinicians to study genetic changes that drive cancer development, progression, and response to treatment, by examining genetic changes at the DNA level. High-throughput sequencing technologies, such as next-generation sequencing (NGS), have revolutionized the ability to identify genetic alterations that drive tumor progression, enabling a fast and cost-effective analysis of the entire genome of cancer cells [22,23]. More specifically, genomic analysis of cancers helps to identify not only specific mutations but also copy number variations and gene expression patterns characteristic of different types of tumors. This information serves as a valuable tool for identifying and better understanding genetic alterations in cancer, and it may also help clarify the intricate biological interplay between different genes. Moreover, the analysis of the genome through genomic studies helps to identify specific biomarkers or molecular characteristics associated with certain types of cancer, which can be used for early detection, risk assessment, and monitoring treatment response. In fact, detailed genomics tumor profiling could aid clinicians in diagnosis, prognosis, and treatment selection for a specific type of cancer. Also, genomic data have led to a more refined classification of cancer types. For example, the classification of lung cancer subtypes through genomic analysis has enabled a more precise subdivision, such as between small-cell lung carcinoma (SCLC) and non-small-cell lung carcinoma (NSCLC), or further into NSCLC subtypes, including adenocarcinoma and squamous cell carcinoma [19,20].

In this regard, genomics plays a fundamental role in the personalized therapy of NSCLC as it enables the identification of specific genetic mutations that drive the selection of the most effective therapeutic approach [24]. The development of targeted therapies could help to address specifically the genetic abnormalities of a patient’s cancer and represents the basis of personalized and precision medicine. These allow for setting up a therapeutic approach tailored to the genetic profile of each specific patient and tumor, representing an important step forward in cancer therapy and leading to more effective treatments with fewer side effects. This has led to the development of personalized therapies targeting mutations such as EGFR and BRAF, as well as rearrangements like ROS1 and ALK, thereby improving treatment efficacy [25,26,27]. For instance, EGFR inhibitors have been highly successful in patients with activating EGFR mutations, highlighting the importance of tailoring treatment based on genetic profiles [28]. Additionally, NSCLC is often complicated by central nervous system (CNS) metastases, which significantly impact patients’ life expectancy and quality of life. Patients with EGFR mutations or ALK rearrangements are particularly prone to CNS involvement, making targeted therapies a potentially more effective treatment option than traditional approaches like radiotherapy or surgery [29,30]. Despite this, clinical trials on treatments such as neurosurgery, radiotherapy, and systemic therapies, either alone or in combination, have shown mixed results. CNS involvement is more common in NSCLC patients with these genetic alterations, emphasizing the need for targeted therapies in upfront treatment, which may potentially replace traditional loco-regional treatments, such as radiotherapy and surgery [30,31]. However, the role of advanced brain imaging techniques, such as Magnetic Resonance Imaging (MRI), in identifying patients who could benefit from local therapies has not been sufficiently explored.

Furthermore, for patients who require concomitant radiotherapy, there is a lack of guidelines on the optimal timing of interventions in combination with precision medicine approaches, such as tyrosine kinase inhibitors, ALK inhibitors, and/or immunotherapies. This highlights the need for further research to integrate better medical and radiation oncology strategies for patients with metastatic NSCLC (mNSCLC) adenocarcinoma and CNS involvement [32,33,34]. Additionally, genetic biomarkers, such as PD-L1 expression, are revolutionizing diagnostics and helping predict responses to immunotherapies targeting the PD-1/PD-L1 axis. The integration of genomics and radiomics is enhancing diagnostic and prognostic capabilities, fostering the development of non-invasive predictive models and improving outcomes for NSCLC patients [35,36].

Tissue biopsy samples are commonly employed in genomics-driven oncology to characterize cancers [37]. However, clinical attention has shifted toward minimally invasive biopsies, aiming to characterize tumors and leverage molecular information helpful for monitoring treatment response, detecting minimal residual disease, and advancing precision oncology. In this regard, liquid biopsy with Next-Generation Sequencing (NGS) represents a revolutionary and promising methodology in the field of oncology [38,39,40]. This innovative approach allows for the collection of fundamental molecular information from samples of biological fluids, such as blood, providing a less invasive alternative to traditional solid tissue biopsies [41]. The use of NGS in liquid biopsy opens the door to in-depth molecular assessment, enabling the analysis of circulating tumor DNA (ctDNA) in the blood [42,43]. This genetic material originates from apoptotic or deceased tumor cells, offering a direct window into the genetic evolution of the tumor over time [44]. A crucial aspect of liquid biopsy with NGS is its ability to identify specific genetic mutations associated with cancer. Through the analysis of oncogenic mutations and the presence of distinctive genetic markers, a detailed understanding of the tumor’s genetic signature can be obtained.

Dynamic monitoring of the tumor is another significant advantage offered by this approach. Liquid biopsy sequenced with NGS allows for real-time tracking of the genetic evolution of the tumor during treatment. This provides a clear insight into the cancer response to therapies and allows for timely adjustment of treatment plans based on emerging genetic mutations. Furthermore, liquid biopsy with NGS facilitates the early detection of recurrences. The presence of ctDNA in the blood can be identified before recurrence becomes apparent through other diagnostic methods, enabling timely intervention [45]. A significant advantage of liquid biopsy is its minimal invasiveness compared to traditional tissue biopsies. This approach significantly reduces discomfort for patients and allows for repeated sampling over time. The application of liquid biopsy with NGS extends to various types of cancer, including breast cancer, lung cancer, colorectal cancer, and many others. In conclusion, liquid biopsy with NGS represents an exciting frontier in oncology, opening new perspectives for dynamic and precise molecular assessment of tumors [46]. This approach promises to guide more targeted and personalized therapies, thereby contributing to the improvement of the management and treatment of oncology patients [47,48,49,50].

In summary, genomics plays a crucial role in treating cancer, enabling personalized treatment strategies and improved diagnostics, while also helping to understand the molecular bases underpinning this complex disease.

## 3. Role of Metabolomics in Cancer

The genomic era of cancer has led to the acknowledgement that certain metabolic enzymes are often altered or amplified in many tumor types. A recent study analyzed a database from The Cancer Genome Atlas, comprising over 10,000 tumors across 32 different cancer types. It identified at least one metabolic gene mutation per tumor, with a diverse array of metabolic gene modifications across different cancer types [51]. For this reason, in this review, we will delve into the most common metabolic alterations found in cancers and the numerous important applications of metabolomics techniques.

Since researchers have recognized that alterations in the genome are not necessarily reflected in the biological phenotype, metabolomics is playing an increasingly important role in cancer research and treatment, offering insights into the metabolic alterations associated with carcinogenesis, cancer development, and therapeutic response. This Omics technique entails the systematic quantification of numerous metabolites, including nutrients, signaling mediators, drugs, and the metabolic byproducts of these small molecules, across a broad variety of biological materials [52]. The most common types of materials used for metabolomics include laboratory-cultured cells, tissues, and tissue extracts, as well as clinical specimens such as tumors, extracts from biopsy or surgical specimens, and body fluids (e.g., blood, plasma, serum, and urine) [9]. Metabolomics represents an emerging discipline and technology in the age of “post genomics” since it provides a functional readout of metabolic processes and thus represents a valuable method for understanding the biochemical basis of cancer. Moreover, since the production of specific metabolites reflects the physiological and pathological state of an organism, metabolomics not only aids in identifying metabolic changes in cancer cells but also provides opportunities for early detection, monitoring therapy response, and the development of novel therapeutic strategies [9,53].

In comparison to genomics and other Omics methodologies, metabolomics possesses distinct properties that can yield a comprehensive assessment of the cumulative modifications occurring at the DNA, RNA, and protein levels and could represent the most sensitive approach to identify pathological alterations. Minor alterations at the genetic level or protein expression are often amplified at the metabolic level, facilitating the detection of physiological and pathological changes in vivo. Like other Omics technologies, metabolomics necessitates a comprehensive database of metabolite information. However, the amount of metabolites in vivo is significantly less than that of whole-genome sequencing data, making the development and improvement of metabolomics databases comparatively straightforward [54,55].

### 3.1. Metabolomics in the Discovery of Cancer Biomarkers in Lung Cancer

Malignant tumors often exhibit abnormal metabolism to support rapid growth and survival, resulting from the disruption of various pathways within the human body [56,57]. Changes in metabolism also affect chromatin dynamics and epigenetic modifications that promote carcinogenesis by altering the availability of substrates necessary for chromatin remodeling [58]. Therefore, metabolomics can help identify the metabolic reprogramming by analyzing metabolites involved in energy production (e.g., glycolysis, oxidative phosphorylation, and the tricarboxylic acid cycle) and biosynthesis (e.g., nucleotides, lipids, and amino acids). In line with these observations, Table 1 summarizes some of the most representative metabolites altered in cancer, including carbohydrates, amino acids, and lipids, highlighting their functional roles and potential clinical significance.

Through the analysis of distinct metabolite patterns in biological fluids (such as blood, urine, or tissue samples), metabolomics allows the identification of possible cancer signatures, representing a promising technology for the identification of both known and new tumor biomarkers for early detection, prognosis, or therapy response monitoring [53,59,60]. Moreover, this approach may aid in selecting tailored treatments that align with the specific metabolic demands of a particular tumor.

An example is given by the alteration of glucose metabolism in cancer cells, which, to support their rapid proliferation, bypass the mitochondrial oxidative phosphorylation used by normal cells for energy supply and utilize glycolysis instead. In fact, in numerous cancer cells, glucose is predominantly metabolized by fermentation to lactate, even in the presence of sufficient oxygen. The conversion of pyruvate into lactate appears to be a crucial process through which cancer cells preserve an optimal equilibrium of redox cofactors, promoting their biological processes [61]. This phenomenon, also known as the “Warburg effect” or aerobic glycolysis, is observed in various tumor types and leads to the accumulation of lactic acid, the most commonly detected metabolite in both blood and tissue samples from oncologic patients [62,63,64]. Multiple signaling pathways altered in cancer could influence glucose metabolism via various mechanisms. For example, receptor tyrosine kinases activated by insulin or other growth hormones initiate the PI3K-AKT signaling pathway to promote glycolysis [65]. Furthermore, growth factor receptor signaling in cancer cells stimulates RAS proteins, facilitating glucose absorption, glycolysis, and the pentose phosphate cycle.

Importantly, mutations in tricarboxylic acid (TCA) cycle enzymes lead to altered metabolite levels in various cancers. For example, germline mutations in two TCA proteins, fumarate hydratase and succinate dehydrogenase, predispose individuals to hereditary cancer syndromes: hereditary leiomyomatosis and renal cell carcinoma, and hereditary paraganglioma-pheochromocytoma, respectively [66,67]. In particular, affected individuals are characterized by the accumulation of high levels of fumarate and succinate respectively, which lead to the inhibition of DNA histone demethylases and prolyl hydroxylases, resulting in DNA hypermethylation and aberrant transcriptional changes responsible for uncontrolled cancer cell growth [68].

Together with glucose, glutamine is also a critical nutrient indispensable for fueling tumor proliferation, growth, and survival, leading to a substantial decrease in serum glutamine levels due to its increased consumption [69,70,71,72,73]. An interesting study conducted by Davidson et al. employed carbon isotopes to label glucose and glutamine in NSCLC, facilitating the analysis of metabolic pathways using isotopic tracers. The research demonstrates that glucose in cancer patients is significantly converted to lactate, with glucose and glutamine identified as the primary carbon sources in the TCA cycle [74]. Furthermore, there is a correlation between cancer aggressiveness and lactic acid levels: specifically, more aggressive cancers exhibit a higher dependency on glycolytic metabolism, resulting in elevated lactic acid concentrations [75,76]. Glutamine also serves as a crucial nitrogen and carbon source for the synthesis of nucleotides, amino acids, and other biochemical pathways that cancer cells utilize to promote the tumorigenesis process [77,78,79]. Moreover, glutamine may also serve as an important biomarker for cancer prognosis: lung cancer patients exhibiting low levels of this amino acid demonstrated shorter survival due to the high requirement of glutamine for cancer cells. In contrast, patients with higher glutamine levels had a more prolonged survival [80]. Furthermore, glutamine is metabolized into glutamic acid and ammonia, which contributes to the pH balance in cancer cells. This process may account for the elevated glutamate levels observed in NSCLC patients [71,80,81]. Glutamate and cysteine are essential components for glutathione (GSH) synthesis. An integrated metabolomic and proteomic study demonstrated that cysteine and glutamic acid concentrations were significantly elevated in lung adenocarcinoma patients when comparing tumor tissues to control tissues [82]. In NSCLC patients, together with glutamine, serum levels of histidine and threonine are also diminished as a result of enhanced utilization of the glycine, serine, threonine, and pyrimidine metabolic pathways [71,83].

Tumor progression is also marked by a notable increase in serum concentrations of lysine, valine, and phenylalanine [71]. In particular, the increased phenylalanine concentration found in tissue samples from lung cancer patients is connected with the down-regulation of proteins related to phenylalanine metabolism, potentially indicating a diminished capacity of cells to metabolize this amino acid in advanced disease stages [84]. Another important cancer biomarker is serine, which plays a crucial role in the metabolism of tumor cells, as it serves as a source of one-carbon units for the folate cycle, essential for the de novo synthesis of purines and thymidine, both of which are necessary for DNA synthesis and cell proliferation. Furthermore, serine has also been demonstrated to contribute to NADPH production [85]. Moreover, the serine synthesis pathway supplies serine for protein synthesis in cancer cells, which, in conjunction with the glycolysis pathway, is an essential metabolic network for cancer. Specifically, the serine synthesis pathway is a significant endpoint for the glycolysis intermediate 3-phosphoglycerate. Consequently, monitoring of serine metabolism is crucial for investigating the onset and progression of lung cancer, as well as enhancing its treatment [86].

Although alterations in glucose and amino acids metabolism are the most commonly observed changes supporting tumor survival, growth, and invasion, lipid dysregulation is also a common feature in oncologic patients. The extensive proliferation of cancer cells requires a substantial increase in lipid hydrolysis, particularly phospholipids, which are essential components of cell membranes. Lipid metabolism also influences the composition and permeability of cell membranes, thus promoting cancer development and progression [87,88]. In this area, important cancer biomarkers are represented by choline and sphingosine, which serve as precursors for phospholipids and sphingolipids. In particular, it has been shown that in comparison to healthy individuals, lung cancer patients exhibit a reduced concentration of choline and sphingosine in serum due to the consumption of malignant tumors to support rapid division and proliferation [71,89]. Also, serum levels of lipoproteins (LDL and VLDL) in lung cancer patients are significantly reduced, which could be related to tumor proliferation, invasion, and metastasis [81]. Other cancer biomarkers for early diagnosis of lung cancer could be represented by sphingosine-1-phosphate (S1P) and ceramides, which are bioactive signaling molecules that regulate pathways central to tumorigenesis. As reported in a nested case–control study, Alberg et al. associated increased plasma levels of these metabolites with a higher risk of lung cancer [90]. In parallel to S1P and ceramides, lysophosphatidylethanolamine (LPE) plays a role in cell signal transduction, which could serve as a diagnostic marker for early-stage lung carcinoma. A recent study demonstrated that LPE concentrations in the plasma of patients with NSCLC were elevated compared to those of healthy controls, particularly in patients with adenocarcinoma [91].

**Figure 2:**
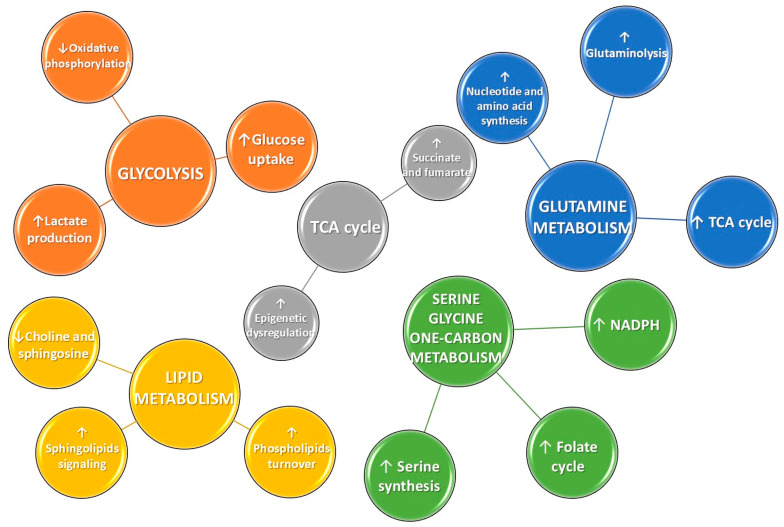
Schematic representation of the most frequently disrupted metabolic pathways in cancer. Upward (↑) and downward (↓) arrows indicate increased or decreased activity, respectively, of the corresponding metabolic pathways or metabolites.

These multiple and interconnected alterations in glucose, amino acid, and lipid metabolism highlight the complex metabolic reprogramming that sustains cancer cell proliferation and survival. To provide a concise overview of these pathways and their main alterations in cancer, the key metabolic changes are summarized in Figure 2.

Notably, metabolomics may facilitate early diagnosis and improved patient outcomes by detecting metabolite alterations before the onset of symptoms. Specific metabolites or metabolic fingerprints might serve as prognostic indicators; for instance, elevated levels of lactate or particular amino acids might correlate with poor prognosis, whereas other metabolites may indicate a better outcome. Especially for the diagnosis of lung cancer, metabolomics is gaining increasing interest in the detection of early and specific biomarkers, and it could efficiently supplement the deficiencies of other techniques, such as imaging examinations. In recent years, low-dose computed tomography (LDCT) has been extensively utilized for the early detection of lung cancer. Compared to chest radiography, LDCT screening can diminish the risk of lung cancer mortality by 20% [92,93,94]. However, LDCT screening may yield a significant number of false-positive results. A prior meta-analysis revealed that in all trials and cohorts, 20% of individuals in each screening phase tested positive and, ultimately, about 1% of patients were diagnosed with lung cancer [95]. Consequently, sensitive and precise biomarkers for lung cancer diagnosis are urgently needed to address the deficiencies of imaging tests.

However, it is important to consider that although metabolomics holds enormous potential for biomarker discovery, early diagnosis, and the characterization of specific biological states, several challenges must be addressed before this technique can be fully integrated into clinical research and routine practice. Covering the entire metabolome requires several complementary approaches, often needing multiple instruments that may not be readily available in academic or small clinical laboratories. A robust experimental design is also necessary for analyzing large metabolomics datasets, which demand adequate statistical analysis. Nevertheless, the field of metabolomics is expanding and is gaining increasing interest in the scientific community, creating new avenues for cancer research.

### 3.2. Metabolomics: Methodology and Instrumentation

In this section, we discuss the general technical features of metabolomics, including the main technologies employed, as well as the benefits and drawbacks of various strategies. More specifically, we will focus on nuclear magnetic resonance (NMR), which is widely and successfully applied for determining metabolites, even from complex samples.

**Figure 3:**
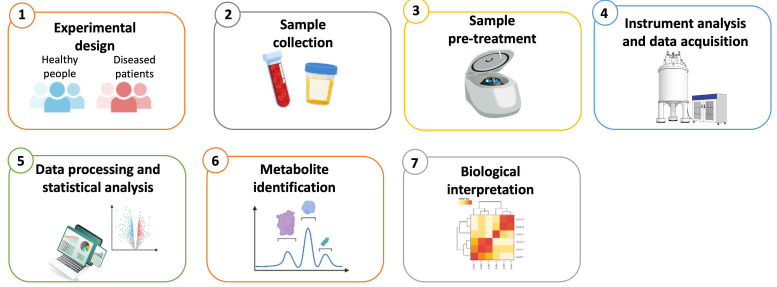
Overview of the key steps in a typical metabolomics workflow. (1) The experimental design defines the hypothesis and methodology, organizes the experimental groups and document all relevant information for each group. (2) Sample collection involves the documentation of detailed information on collected sample and the selection of appropriate storage conditions. (3) Sample pre-treatment involves the application of suitable preparation procedures based on the sample type and the instrument platform used. (4) Instrument analysis and data acquisition involve three main metabolomics platforms: LC-MS, GC-MS, and NMR spectroscopy. (5) Statistical methods include t-test, ANOVA, PCA, PLS-DA, and OPLS-DA. Clustering and visualization tools generate dendrograms and heatmaps. (6) Metabolites are identified and characterized through database matching. (7) The final biological interpretation involves the screening for biological markers and evaluation of correlated metabolic pathways.

In practice, metabolomics analyzes small molecule metabolites (≤1500 Daltons) in a biological material. It entails the concurrent identification of multiple compounds, primarly based on the chemical characteristics and/or atomic weights of the molecules. The field of metabolomics has significantly benefited from recent technological advancements in instrumentation, resulting in more cost-effective devices with a reduced footprint. Intuitive software and large datasets have facilitated the rapid and precise processing of data. Additionally, numerous open-source software programs are available for analyzing metabolomics data [96]. In general, all metabolomics investigations typically involve several steps, including sample collection and pre-treatment, instrumental analysis, and data processing (Figure 3).

Several approaches can be considered for this analysis, each with its own advantages and disadvantages. Determining whether to use a targeted or untargeted approach is the first step in selecting the most effective method. More specifically, targeted metabolomics is utilized when testing a particular hypothesis or during the validation and implementation phases, and it is based on the identification and quantification of a limited number of known metabolites to be measured prior to conducting the analysis. Hence, the targeted approach represents the best option if the target metabolites to be investigated have already been identified [97].

On the other hand, the untargeted approach aims to acquire data for as many species as possible, and it is typically used for hypothesis generation and widely applied for the discovery of new biomarkers. Most researchers typically use untargeted metabolomics when investigating disease biomarkers, as it is a comprehensive and unbiased detection method that enables a global profiling of the metabolome [98]. It is essential to note that while targeted metabolomics provides accurate quantitative analysis by establishing standards and isotopes, untargeted metabolomics can only achieve relative quantitation based on the response intensity of signal peaks.

Metabolomics investigates a complex and chemically varied group of compounds (sugars, lipids, and other organic compounds) featuring diverse physical and chemical properties; therefore, their examination could require a number of sample preparation and data collection techniques. As a result, the analysis of data obtained through metabolomics experiments requires remarkable expertise and effort, especially for untargeted metabolomics, whose aim is to identify unknown metabolites [98]. While other omics techniques (such as genomics, transcriptomics, and proteomics) focus on molecules and macromolecules with limited chemical diversity and well-defined structures, making them suitable for a single methodology to obtain a comprehensive analysis, metabolomics deals with a vast and heterogeneous array of small molecules. This chemical complexity requires the use of multiple, complementary analytical methods to capture the full spectrum of metabolites present in a biological system.

In light of this, it is clear that the importance of the technology applied to metabolomics studies lies in aiding the biological understanding and interpretation of complex data. The primary methods used for metabolomics are mass spectrometry (MS) and nuclear magnetic resonance (NMR), both of which are suitable for detecting compounds in the liquid phase of any biofluid or cell/tissue extract. NMR is also applicable to solid-phase samples, including cells, tissues, and cell membranes. There are also other methods based on these techniques able to analyze metabolites in situ, including matrix-assisted laser desorption/ionization mass spectrometric imaging (MALDI-MSI) and NMR-based in vivo imaging, like magnetic resonance spectroscopic imaging (MRSI), that will be not further discussed in this review, therefore we refer the reader to several manuscripts for more comprehensive coverage of these topics [99,100,101,102].

### 3.3. Nuclear Magnetic Resonance (NMR) Metabolomics Applications in NSCLC

Nuclear magnetic resonance (NMR) is a technique commonly used for determining the molecular structure, composition, and purity of a specific sample. The possibility to employ NMR with biospecimens in the liquid, solid, or gas phase, without the need for complicated sample pre-processing, makes it a versatile technique. Importantly, this technique is the only metabolomics platform available for in vivo detection. Moreover, it provides a quantitative analysis of metabolite concentration, and it features simple analytical conditions, good repeatability, easy quantification, and high objectivity. Other analytical methods necessitate laborious calibrations, chemical derivatization, and extensive sample preparation.

In practice, NMR analysis consists of submitting a sample to radiofrequency waves after it has been exposed to a strong magnetic field. When aligned in a strong magnetic field, specific nuclei in a molecule (such as ^1^H, ^13^C, ^15^N, or ^31^P) are transiently excited by radiofrequency radiation, causing a switch in their spin state. This nuclei relaxation results in a distinctive chemical shift that indicates the type, location, and electromagnetic environment of the excited atoms in that specific molecule. NMR is one of the first-choice techniques used in metabolomics, as the sample is not consumed or destroyed during the analysis, allowing for further studies. This differs from MS approaches, where the material is entirely consumed during the process. However, the main disadvantages of NMR are its large footprint and sensitivity in the micromolar range, compared to mass spectrometry, which has a sensitivity in the nanomolar range [103]. Moreover, the diversity of chemical species that NMR can feasibly measure is also less than that of MS-based approaches, because the majority of metabolites are relatively low-abundance in biological materials. However, NMR techniques continue to evolve and improve. To enhance the sensitivity of NMR procedures, cryogenic probes and microprobes are commonly used, as they increase signal strength by boosting magnetic field intensity [104,105]. Importantly, in the last few years, high-resolution magic angle spinning (HRMAS) magnetic resonance spectroscopy (MRS) has been widely used in metabolomics for the direct detection of non-liquid tissue and cell specimens. In fact, this technique has become one of the major platforms in metabolomics studies, as it preserves the cellular structure of tissues—making it suitable for pathological evaluations—while also producing high-resolution spectra comparable to those obtained from solution-based extracts, without requiring complicated metabolite extraction procedures. Moreover, due to its high-resolution performance, it requires small sample amounts, thus representing a non-invasive technique and an excellent tool for studying metabolism, offering a more accurate understanding of the metabolic patterns of cancers [106,107].

Many studies conducted over the past few years have shown encouraging outcomes in characterizing the metabolic profiles of lung cancer patients. In 2016, Puchades-Carrasco et al. [71] analyzed the serum metabolic profile of NSCLC patients at different stages using ^1^H-NMR and they compared it with that of healthy individuals and patients diagnosed with benign pulmonary diseases. The authors reported lower levels of HDL, LDL, VLDL, choline, glucose, glutamine, threonine, and histidine in patients with NSCLC. In comparison, significantly higher levels of lactate, leucine/isoleucine, *N*-acetyl-cysteine, glutamate, and creatine were detected. Moreover, they found a correlation between disease progression and an increase in the concentrations of lysine, valine, and phenylalanine (Table 2) [71]. In parallel, Zhang et al. analyzed the changes in serum metabolite levels in lung cancer patients employing both non-targeted metabolic profiling based on ^1^H-NMR and a targeted approach based on rapid resolution liquid chromatography (RRLC). The main metabolic alterations included an increase in serum ketone bodies (β-hydroxybutyrate and acetoacetate) and lactate, with a decrease in glucose, lipoproteins (LDL and VLDL), unsaturated lipids, choline metabolites (glycerophosphocholine, phosphocholine, and choline), trimethylamine *N*-oxide (TMAO), and betaine. Moreover, lung cancer patients had higher serum levels of the majority of amino acids, including glutamine, glutamate, asparagine, aspartate, histidine, tyrosine, isoleucine, leucine, and cysteine. Conversely, these patients had lower levels of methionine and tryptophan (Table 2) [81]. Two years later, Hu et al. aimed to identify potential serum biomarkers for evaluating microwave ablation (MWA) treatment in NSCLC using ^1^H NMR-based metabolomics. In their study, the authors collected serum samples from 43 healthy individuals, 39 patients with advanced-stage NSCLC, and 38 NSCLC patients who had undergone MWA. They subsequently used NMR to evaluate the samples and examined the variations in metabolites through ^1^H NMR-based analysis. The outcomes showed that patients with NSCLC had significantly higher serum levels of lactic acid, glutamate, tryptophan, alanine, phenylalanine, tyrosine, proline, and glycoprotein. However, their levels of glucose, glutamine, glycine, and taurine were lower than those of healthy people (Table 2). Moreover, after MWA treatment, the metabolic profiles of NSCLC patients shifted toward those of the healthy controls. The findings suggest that ^1^H NMR metabolomics can improve understanding of NSCLC pathophysiology and demonstrate the therapeutic impact of MWA. The altered metabolites identified may serve as potential biomarkers for the diagnosis of NSCLC and for monitoring the efficacy of MWA treatment [108]. Serum samples were also analyzed in a recent study by Schult et al. to quantify variations in metabolites between two groups employing high-resolution magic angle spinning proton magnetic resonance spectroscopy (HRMAS ^1^H NMR). In particular, they collected samples from 79 patients with NSCLC and 79 healthy participants. They found that glycolysis and the tricarboxylic acid cycle metabolism were the metabolic pathways most clearly impacted by lung cancer. Moreover, they detected alterations in vitamins, coenzymes, organic acids, amino acids, carnitine, sugar phosphates, nucleosides, and nucleobases that could lead to a model for both the early detection and the prediction of the 5-year survival rate of lung cancer patients, demonstrating the potential of serum metabolomics as a non-invasive tool (Table 2) [109]. Another important application of HRMAS ^1^H NMR was presented in a 2017 study by Chen et al. [110], who collected and analyzed lung cancer tissue samples from 32 patients. In more detail, they examined the NMR metabolomic fingerprints combined with multivariate data analysis (MVDA). They found that the levels of lipids, choline-containing compounds (i.e., glycerophosphocholine and phosphocholine), and aspartate in tumor tissues changed significantly at different stages, highlighting the potential of this technique as a non-destructive tool in cancer diagnosis and staging, which complements traditional imaging and histopathology (Table 2) [110]. In another study, Berker et al. employed HRMAS MRS to analyze plasma and tissue samples from 93 patients with NSCLC and healthy controls. Researchers applied multivariate statistical models to identify metabolic biomarkers that could distinguish between the two main NSCLC subtypes (adenocarcinoma and squamous cell carcinoma), as well as to assess disease staging and predict patient survival (Table 2) [80]. NMR metabolomics was also employed in 2021 by Sarlinova et al. [111] to plasma samples from 132 patients with primary lung cancer and 47 with secondary lung cancer. The obtained outcomes showed that the two patient groups did not differ significantly in their plasma metabolites. Moreover, both lung cancer groups reported significantly higher levels of glucose, acetate, creatinine, citrate, and 3-hydroxybutyrate, and lower levels of lactic acid, pyruvate, tyrosine, alanine, and tryptophan when compared to healthy people (Table 2) [111]. In the same year, Ahmed et al. analyzed 32 pairs of serum samples and 29 pairs of urine samples from patients with early-stage NSCLC using NMR and mass spectrometry, both before and after surgery. The findings demonstrated that patients with lung cancer exhibited greater metabolic changes in urine than in blood, both before and after surgery. Specifically, after surgery, the levels of isopentenyladenine, leucyl-proline, fumaric acid, and asymmetric dimethylarginine (ADMA) in urine samples dramatically dropped. In particular, isopentenyladenine decreased to 1/625 of its pre-surgery level, while leucyl proline decreased to 1/31 of its pre-surgery level. Nevertheless, following surgery, the concentration of *N*^6^-methyladenosine, which has a critical role in the oncogene regulation and pathogenesis of several human cancers (including NSCLC), rose to 27 times its pre-operation level (Table 2). The metabolic biomarkers found in this study could serve as a basis for tracking the effects of treatment and potentially for determining whether lung cancer is present or absent [112]. Another important study, based on the combination of NMR and mass spectrometry, was conducted by Seow et al., who applied an untargeted metabolomic analysis to identify metabolites and pathways associated with a decreased risk of lung cancer. Specifically, in their case–control study, they analyzed urine samples from 275 never-smoking women with lung cancer and 289 never-smoking healthy women of comparable age. The study’s findings revealed a protective correlation between the incidence of lung cancer and 5-methyl-2-furoic acid (Table 2), which is a metabolite associated with dietary soy consumption. The authors hypothesized that some bioactive substances found in soy would possess anti-inflammatory and antioxidant properties, which could prevent tumor development and invasion, and trigger apoptosis [113].

In summary, NMR metabolomics analysis gives an important contribution to the discovery of lung cancer biomarkers that hold great potential as tools for mechanistic studies, disease screening, diagnosis, and prognosis. Moreover, the ongoing expansion of diverse metabolomics databases, the improvement of detection instruments’ performance, and the spreading of machine learning offer a more reliable way to identify variations in metabolites effectively. Finally, in the last year, multi-omics joint analysis has emerged as a new research hotspot due to the constant and productive collaboration between genomics, transcriptomics, proteomics, and metabolomics [3,114]. In this way, researchers can acquire more comprehensive biological information for in-depth mechanistic study of specific diseases.

## 4. Role of Radiomics in Lung Cancer

**Figure 4:**
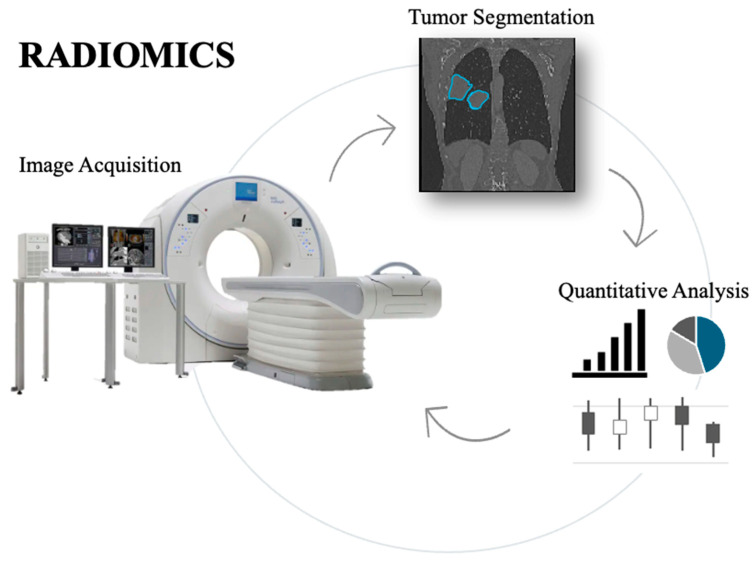
Overview of the radiomics workflow, showing the process from medical image acquisition to tumor segmentation, extraction of key quantitative features, and their analysis for potential clinical applications.

Radiomics is a rapidly evolving discipline that specializes in extracting and quantifying tumor characteristics, including intensity, shape, and texture, from medical images. This technique permits a detailed analysis of tumor phenotypes, presenting complementary and impartial records that may manual customized treatment strategies. By supplying a deeper understanding of tumor biology, radiomics can help clinicians more accurately assess therapeutic effectiveness and tailor treatments accordingly. However, as radiomics is still in development, its full capacity to enhance diagnostic, prognostic, and predictive accuracy in clinical practice can only be determined through continuous improvement, validation, and committed funding [115,116]. Radiomics is an innovative technique that employs quantitative analysis of medical images to extract detailed features crucial for tumor diagnosis and management. By leveraging advanced algorithms, radiomics transforms traditional imaging techniques—such as computed tomography (CT), magnetic resonance imaging (MRI), and positron emission tomography (PET)—into rich data that offers predictive insights into tumor pathology. Unlike conventional methods that mainly focus on tumor size and shape, radiomics can detect subtle variations in tumor texture, shape, and distribution, providing a more comprehensive understanding of tumor heterogeneity, subtype differentiation, and clinical outcomes [117,118]. The radiomics methodology consists of several key steps: image acquisition, segmentation of regions of interest (ROIs), feature extraction, and statistical correlation with clinical outcomes (Figure 4). A recent advancement in the field, “delta radiomics,” tracks changes in radiomic features over time, enabling a more accurate assessment of tumor evolution and response to specific therapeutic interventions [119]. Tools such as Pyradiomics, which adhere to the Imaging Biomarker Standardization Initiative (IBSI) guidelines, have standardized the extraction of features, including 3D, first-order, and second-order features (e.g., GLCM and GLRLM). This standardization has enhanced the reproducibility and reliability of radiomics studies, thereby increasing their applicability in clinical practice [118,119,120].

Radiomics features are classified into various categories, each providing unique insights into tumor characteristics:

1. First-Order Features: These features analyze the distribution of intensities within the ROI, without considering the spatial relationship between voxels. They provide information on the variability of tumor intensity and its homogeneity. Common first-order features include:
  - Mean: the average intensity of the voxels within the ROI.
  - Standard Deviation: a measure of the dispersion of intensities.
  - Skewness: the symmetry of the intensity distribution.
  - Kurtosis: a measure of the shape of a distribution.
  - Entropy: a measure of the complexity and disorder of the intensity.
2. Second-Order Features (Texture Features): These features focus on the spatial relationships between voxels, describing the texture and heterogeneity of the tumor. Common techniques used to extract these features include gray-level co-occurrence matrices (GLCMs) and gray-level run-length matrices (GLRLMs). Key second-order features include:
  - Contrast: a measure of the difference in intensity between adjacent voxels.
  - Correlation: a measure of the correlation between the intensities of voxels.
  - Energy: a measure of the uniformity of intensity distribution.
  - Homogeneity: a measure of the uniformity of intensities.
3. Third-Order Features (Shape Features): These features describe the geometry of the ROI, such as the shape and size of the tumor. Some examples include:
  - Volume: the total volume of the region of interest.
  - Sphericity: describes how close the shape of the ROI is to a sphere.
  - Surface Area: the surface area of the ROI.
  - Compactness: the ratio of volume to surface area, describing the regularity of the shape.
4. Higher-Order Statistics: These features focus on advanced measures, such as the fractal dimension of the tumor surface, which provides information on its irregularity and geometric complexity. An example is:
  - Fractal Dimension: a measure of the complexity of the tumor’s geometry, useful for characterizing more irregular tumors.

By combining these diverse categories of features, radiomics offers a detailed characterization of tumors, enhancing diagnostic and prognostic processes and enabling more personalized patient management. When integrated with clinical and genetic data, radiomics can significantly improve diagnostic accuracy and inform therapeutic strategies.

Moreover, combining radiomics data with other omics disciplines (e.g., genomics, proteomics) enables a deeper understanding of cancer biology, ultimately guiding personalized treatment approaches. This is especially important for lung cancer, the deadliest form of cancer, with the highest mortality rate [14,121,122].

The applications of radiomics in lung cancer concern the early diagnosis of the primary tumor, prognostic assessment, identification of metastatic lesions, radiogenomic profiling of the primary tumor, and prediction of the response to therapy [123,124,125].

Concerning the diagnosis of the primary tumor, Zhang et al. review the application of radiomics combined with dual energy CT in early detection and treatment decision-making. The included paper aimed to perform a quantitative analysis on multiple features [126]. The differential diagnosis between benign and malignant pulmonary nodules achieved an AUC of 0.877 by combining DECT with radiomics analysis. These results can provide patients with the earliest diagnosis, dose-sparing, and a reduction in health-care costs.

Several other studies focused on treatment strategies, studying the molecular profile of the tumor. Wang et al. predict the presence of mutations of EGFR and TP53 in 267 lung adenocarcinomas. They achieve an AUC of 0.811 when the radiomic model is merged with the clinical model. The model could identify the TKI responders and those with poor prognosis [127]. In the study by Kirienko et al., radiomic analysis was performed on 151 patients using both PET/CT and CT images to predict gene expression profiles and identify histotype, aggressiveness, and progression [128]. The best-performing rule in outcome prediction for 73 cases, which included the entire set of imaging exams, KRAS/TP53/EGFR mutational status, and 238 differentially expressed genes, was 0.87.

The diagnosis of lung cancer from indeterminate pulmonary nodules (IPNs) remains particularly challenging. In a recent multi-institutional study involving 2032 participants with IPNs, clinical data, radiomic features, and fragmentomic characteristics of circulating cell-free DNA in 5-methylcytosine (5mC)-enriched regions were integrated to construct a multi-omics model (clinic-RadmC) capable of predicting malignancy risk. The clinic-RadmC model achieved an AUC of 0.923 in the external test set, significantly outperforming single-omics models as well as those combining only clinical and radiomic or fragmentomic features in 5mC-enriched regions (*p* < 0.050 for all). In summary, this study highlights how clinic-RadmC provides a more effective and non-invasive tool for optimizing lung cancer diagnosis, thereby facilitating precision interventions [129].

Recent advances highlight the role of radiogenomics in precision oncology. For instance, Ryan et al. reviewed how integrating imaging and genomic data is reshaping the management of oncogene-driven lung cancer. Their work illustrates that radiogenomic models can non-invasively predict actionable mutations such as EGFR, KRAS, and ALK, support treatment selection, and monitor resistance development, thereby reducing the need for repeat biopsies. Although still experimental, this approach underscores the potential of radiogenomics as a “virtual biopsy” and a future cornerstone of personalized cancer care [130]. Finally, radiomics, when combined with transcriptomic, proteomic, and metabolomic data, has the potential to generate highly predictive models for lung cancer diagnosis and treatment [131]. This integrative approach leverages imaging-derived features alongside molecular signatures, enabling non-invasive insights into tumor biology and patient-specific heterogeneity. Recent studies have demonstrated that such multimodal models can improve histological classification, identify actionable mutations, and predict therapy response with greater accuracy than single-modality analyses. By bridging radiomics with multi-omics, these predictive frameworks pave the way toward more reliable stratification tools and personalized therapeutic strategies in precision oncology [131].

## 5. Conclusions and Future Directions

The integration of multi-omics technologies is reshaping the clinical management of lung cancer, enabling more advanced diagnostics and truly personalized treatment strategies. Genomic profiling has uncovered actionable mutations—such as EGFR, ALK, ROS1, and BRAF—that guide targeted therapies and immunotherapy decisions based on biomarkers like PD-L1 expression. Liquid biopsy, combined with next-generation sequencing (NGS), allows for real-time, minimally invasive monitoring of tumor evolution and the detection of minimal residual disease. Metabolomics, particularly through NMR-based platforms, provides valuable insights into tumor metabolism, helping to identify biomarkers relevant for diagnosis, prognosis, and therapy monitoring. Radiomics complements these approaches by extracting quantitative imaging features that reflect tumor heterogeneity and help predict clinical outcomes.

**Figure 5:**
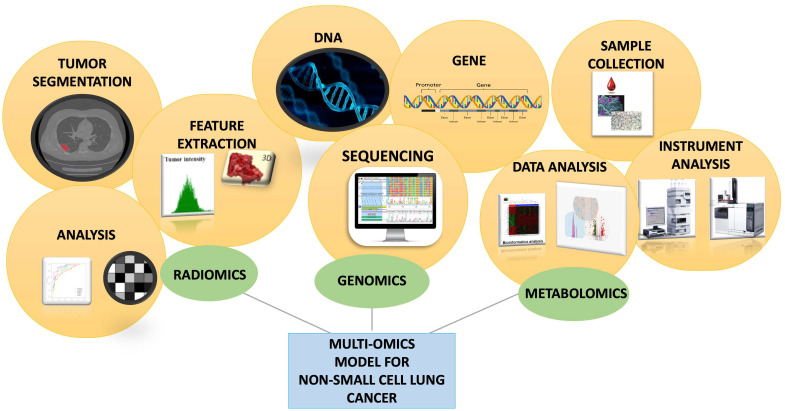
Schematic representation of the integration of radiomics, genomics, and metabolomics in a multi-omics model for non-small cell lung cancer.

As shown in Figure 5, the multi-omics model for non-small-cell lung cancer integrates three complementary domains. Radiomics starts with tumor segmentation on imaging, followed by feature extraction and quantitative analysis of tumor intensity, shape, and heterogeneity. Genomics relies on DNA and gene-level investigations through sequencing technologies, uncovering clinically relevant mutations and guiding targeted therapies. Metabolomics begins with sample collection and high-throughput instrument analysis, generating complex datasets that undergo bioinformatics-driven interpretation to reveal metabolic signatures of disease. Taken together, these parallel pipelines highlight how radiomics, genomics, and metabolomics provide distinct and synergistic insights.

**Figure 6:**
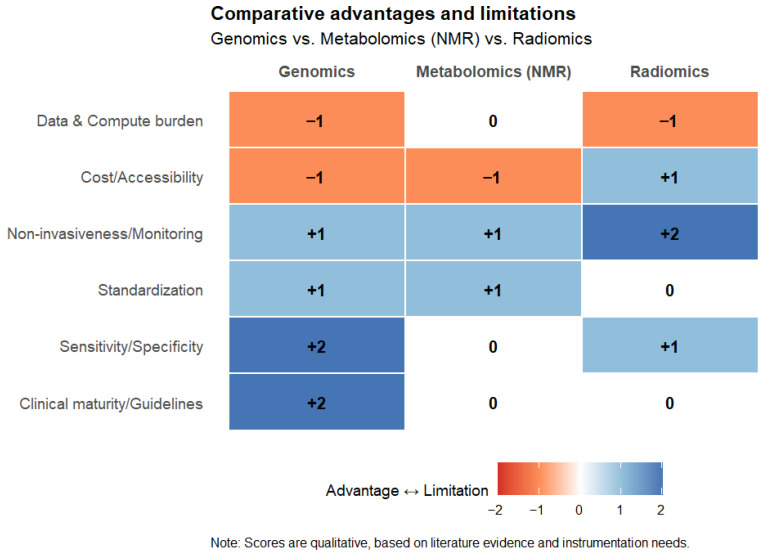
Comparative heatmap summarizing the advantages (+) and limitations (−) of genomics, metabolomics (NMR-based), and radiomics across key evaluation domains, including clinical maturity, sensitivity/specificity, standardization, non-invasiveness, cost/accessibility, and computational burden. Scores were qualitatively assigned based on the literature. The heatmap was created using RStudio R4.5.1.

A comparative heatmap in Figure 6 summarizes the advantages and limitations of genomics, metabolomics, and radiomics across key evaluation dimensions. This visual overview highlights the heterogeneity of these approaches, the advanced instrumentation required, and the complementary nature of the information they deliver, thereby underlining the importance of their integrative use in precision medicine.

Despite these progresses, several challenges must be addressed for full clinical integration. Particularly, standardization of protocols for sample preparation, data acquisition, and analysis is essential to ensure reproducibility. Biomarker signatures must be prospectively validated in large, diverse patient cohorts to confirm their clinical utility. Importantly, the development of advanced bioinformatics tools and machine learning algorithms will be critical to integrating multi-omics data into actionable, decision-support systems. Therefore, multidisciplinary collaboration—among oncologists, radiologists, pathologists, bioinformaticians, and regulatory authorities—is vital to overcoming these hurdles. Ultimately, the convergence of genomics, metabolomics, and radiomics holds great promise for advancing precision oncology and improving outcomes for patients with lung cancer.

## Figures and Tables

**Table 1:** Summary of representative metabolites whose levels are commonly altered in cancer.

Metabolite	Alteration in Cancer	Role/Significance
Glucose	Decreased serum levels due to high cellular uptake and consumption	Energy source; Converted by fermentation to lactate; Supports rapid proliferation.
Lactate	Increased intracellular levels due to enhanced glucose metabolism	Marker of increased glycolytic metabolism; Correlates with cancer aggressiveness.
Glutamine	Decreased intracellular levels due to enhanced glutaminolysis	Carbon/nitrogen source for nucleotide and amino acid synthesis; Supports rapid proliferation.
Glutamate	Increased intracellular levels due to enhanced glutaminolysis	Derived from glutamine; Contributes to pH balance; Essential for glutathione biosynthesis, protecting cells from oxidative stress.
Cysteine	Increased intracellular level due to high cellular uptake and biosynthesis	Essential for glutathione biosynthesis, protecting cells from oxidative stress; Provide sulfur for proteins and cofactors biosynthesis.
Histidine	Decreased intracellular levels due to enhanced utilization	Utilized in glycine, serine, threonine, and pyrimidine pathways; Employed for protein biosynthesis; Precursor of histamine.
Threonine	Decreased intracellular levels due to enhanced utilization	Provides one-carbon units for nucleotide biosynthesis and tricarboxylic acid cycle; Employed for protein biosynthesis.
Serine	Decreased intracellular levels due to enhanced utilization	Source of one-carbon units for folate cycle; Employed for protein and nucleotide biosynthesis; Involved in NADPH production.
Choline	Increased intracellular levels due to enhanced uptake	Precursor for phospholipids; Enhances cell membrane biosynthesis; Supports rapid proliferation.
Sphingosine	Decreased intracellular levels due to enhanced utilization	Conversion in sphingosine-1-phosphate (S1P); Precursor for sphingolipids; Involved in membrane structure.
Sphingosine-1-phosphate (S1P)	Increased intracellular levels due to enhanced sphingosine conversion	Bioactive lipid promoting tumor growth, survival and invasion.
LDL/VLDL	Increased intracellular levels due to enhanced cellular uptake	Supply cholesterol and fatty acids for membrane biosynthesis; Provides energy for tumor metabolism.

**Table 2:** Application of NMR-based metabolomics to identify metabolite alterations in lung cancer.

Patient Groups	Sample Types	Results Compared to Healthy Controls	Reference
NSCLC; HC	Serum	(+) Lactate, leucine/isoleucine, *N*-acetyl-cysteine, glutamate, creatine, acetate, glicerol (−) HDL, LDL, VLDL, choline, glucose, glutamine, threonine, histidine, adipic acid	[71]
93 NSCLC; 29 HC	Serum, tissue	(+) Lactate, glutamate (−) Glycerophosphocholine	[80]
25 LC; 25 HC	Serum	(+) β-hydroxybutyrate, acetoacetate, lactate, glutamine, glutamate, asparagine, aspartate, histidine, tyrosine, isoleucine, leucine, cysteine (−) Glucose, LDL, VLDL, unsaturated lipids, glycerophosphocholine, phosphocholine, choline, trimethylamine *N*-oxide, betaine, methionine, tryptophan	[81]
39 NSCLC; 43 HC	Serum	(+) Lactate, proline, tyrosine, phenylalanine, alanine, tryptophan, glutamate, glycoprotein (−) Glucose, taurine, glutamine, glycine, threonine, phosphocreatine	[108]
79 NSCLC; 79 HC	Serum	(+) ADP, AMP, lactate, fructose-6-phosphate, diphospho-glycerate, 3-phosphoglycerate, tryptophan, succinate (−) ATP, GTP, NADP, 1,7-Dimethyl-xanthine, carnosine, carnitine, taurine, tyrosine	[109]
32 LC	Tissue	(+) Lipids, aspartate, glycerophosphocholine, phosphocholine	[110]
132 PLC; 47 SLC; 77 HC	Plasma	(+) Glucose, acetate, citrate, creatinine, 3-hydroxybutyrate, proline (−) Lactate, pyruvate, succinate, tyrosine, alanine, tryptophan, threonine, lipoproteins	[111]
29 NSCLC urine; 32 NSCLC serum (sampling before and after surgery)	Urine, serum	(+) *N*^6^-methyladenosine (−) Leucyl proline, isopentenyladenine, fumaric acid, ADMA	[112]
275 LC; 278 HC	Urine	(−) 5-methyl-2-furoic acid	[113]

Abbreviations: HC = Healthy Control, NSCLC = Non-Small-Cell Lung Cancer, LC = Lung Cancer, PLC = Primary Lung Cancer, SLC = Secondary Lung Cancer. (+) = increased levels; (−) = decreased levels.

## Data Availability

No new data were created or analyzed in this study. Data sharing is not applicable to this article.

## Abbreviations

The following abbreviations are used in this manuscript:

NSCLC	Non-small-cell lung cancer
SCLC	Small-Cell Lung Cancer
LC	Lung Cancer
CT	Computed Tomography
MRI	Magnetic Resonance Imaging
US	Ultrasound
NGS	Next-generation Sequencing
CNS	Central nervous system
ctDNA	Circulating tumor DNA
FDG-PET	^18^F-deoxyglucose-positron emission tomography
GLUT	Glucose transporter
mTORC1	Mammalian target of rapamycin complex 1
HIF	Hypoxia-inducible factor
HK	Hexokinase
PDAC	Pancreatic ductal adenocarcinoma
TCA	Tricarboxylic acid
GSH	Glutathione
S1P	Sphingosine-1-phosphate
LPE	Lysophosphatidylethanolamine
LDCT	Low-dose computed tomography
NMR	Nuclear magnetic resonance
MS	Mass spectrometry
MALDI-MSI	Matrix-assisted laser desorption/ionization mass spectrometric imaging
MRSI	Magnetic resonance spectroscopic imaging
HRMAS MRS	High-resolution magic angle spinning magnetic resonance spectroscopy
RRLC	Rapid resolution liquid chromatography
TMAO	Trimethylamine *N*-oxide
MWA	Microwave ablation
MVDA	Multivariate data analysis
ADMA	Asymmetric dimethylarginine
HC	Healthy control
PLC	Primary lung cancer
SLC	Secondary lung cancer
ROI	Region of interest
IBSI	Imaging Biomarker Standardization Initiative
GLCM	Gray-level co-occurrence matrices
GLRLM	Gray-level run-length matrices
